# Randomized double-blind placebo-controlled trial of 40 mg/day of atorvastatin in reducing the severity of sepsis in ward patients (ASEPSIS Trial)

**DOI:** 10.1186/cc11895

**Published:** 2012-12-11

**Authors:** Jaimin M Patel, Catherine Snaith, David R Thickett, Lucie Linhartova, Teresa Melody, Peter Hawkey, Anthony H Barnett, Alan Jones, Tan Hong, Matthew W Cooke, Gavin D Perkins, Fang Gao

**Affiliations:** 1 Academic Department of Anaesthesia, Pain and Critical Care, Heart of England NHS Foundation Trust, Bordesley Green East, Birmingham, B9 5SS, UK; 2 School of Clinical and Experimental Medicine, University of Birmingham, Edgbaston, Birmingham, B15 2TT, UK; 3 Department of Public Health and Bacteriology, University of Birmingham, Edgbaston, Birmingham, B15 2TT, UK; 4 Department of Microbiology, Heart of England NHS Foundation Trust, Bordesley Green East, Birmingham, B9 5SS, UK; 5 Diabetes Centre, Heart of England NHS Foundation Trust, Bordesley Green East, Birmingham, B9 5SS, UK; 6 Department of Pathology, Heart of England NHS Foundation Trust, Bordesley Green East, Birmingham, B9 5SS UK; 7 Health Sciences, Medical School Building, Gibbet Hill Campus, University of Warwick, Coventry, CV4 7AL, UK; 8 Department of Accident & Emergency, Heart of England NHS Foundation Trust, Bordesley Green, Birmingham, B9 5SS, UK; 9 Clinical Trials Unit, Warwick Clinical Trials Unit, Warwick Medical School, University of Warwick, Coventry, CV4 7AL, UK

## Abstract

**Introduction:**

Several observational studies suggest that statins modulate the pathophysiology of sepsis and may prevent its progression. The aim of this study was to determine if the acute administration of atorvastatin reduces sepsis progression in statin naïve patients hospitalized with sepsis.

**Methods:**

A single centre phase II randomized double-blind placebo-controlled trial. Patients with sepsis were randomized to atorvastatin 40 mg daily or placebo for the duration of their hospital stay up to a maximum of 28-days. The primary end-point was the rate of sepsis progressing to severe sepsis during hospitalization.

**Results:**

100 patients were randomized, 49 to the treatment with atorvastatin and 51 to placebo. Patients in the atorvastatin group had a significantly lower conversion rate to severe sepsis compared to placebo (4% vs. 24% p = 0.007.), with a number needed to treat of 5. No significant difference in length of hospital stay, critical care unit admissions, 28-day and 12-month readmissions or mortality was observed. Plasma cholesterol and albumin creatinine ratios were significantly lower at day 4 in the atorvastatin group (p < 0.0001 and p = 0.049 respectively). No difference in adverse events between the two groups was observed (p = 0.238).

**Conclusions:**

Acute administration of atorvastatin in patients with sepsis may prevent sepsis progression. Further multi-centre trials are required to verify these findings.

**Trial Registration:**

International Standard Randomized Control Trial Registry ISRCTN64637517.

## Introduction

Sepsis describes a complex clinical syndrome that results from a harmful or damaging host response to infection. It is commonly seen in hospital emergency departments and wards with an estimated prevalence of 15% in the UK. A significant proportion of patients with sepsis go on to develop severe sepsis or septic shock with associated mortality rates of up to 50% [1,2]. Despite considerable research there still remains a lack of targeted pharmacological interventions to treat and improve outcomes from sepsis.

Statins inhibit 3-hydroxy-3 methylglutaryl coenzyme A (HMG-CoA) reductase, a rate-limiting enzyme in the biosynthesis of cholesterol. They are well established in the prevention of cardiovascular disease and have been shown to exert numerous effects in addition to their lipid-lowering properties. These pleiotropic properties include anti-inflammatory and immunomodulatory effects resulting in improved endothelial function, reduced thrombogenicity and plaque stabilisation [3-5].

The inflammatory and immune response provoked by sepsis could potentially be modulated by statins via these pleiotropic effects. Several systematic reviews have concluded that statins have a role in improving infection-related outcomes and mortality but most of this evidence comes from retrospective and prospective observational studies [6,7]. In a prospective cohort study of 361 patients it was reported that patients already receiving statin therapy had reduced rates of severe sepsis and intensive care admissions [8]. So far there have been only two published randomized controlled trials (RCTs) investigating the role of statins in sepsis. One of these, a double-blind RCT to assess whether acute administration of statins reduced sepsis progression and cytokine production was stopped prematurely due to slow recruitment and was unable to analyse data relating to sepsis conversion. There were significantly decreased levels of IL-6 and TNF-α at 72 hours, however, compared with baseline (*P *= 0.02) [9]. A more recent RCT investigated whether continuation of pre-existing statin therapy prevented conversion of sepsis to severe sepsis. It concluded that continuation of statin therapy did not reduce progression of sepsis or the development of organ failure and that the witholding of statin therapy did not result in any inflammatory rebound [10].

The recent withdrawal from the market of recombinant activated protein C (drotrecoginalfa, rhAPC) following the results of the PROWESS-Shock trial has resulted in there being no specific therapy for sepsis other than source control and antibiotics [11,12]. Statins offer a safe multi-modal mechanism to target the inflammatory and immunological cascade of sepsis which could be prescribed to patients prior to the establishment of septic shock.

The aim of this study was to evaluate whether the acute use of atorvastatin 40 mg/day in ward patients with sepsis would significantly reduce rates of conversion of sepsis to severe sepsis (≥ one organ failure) compared with placebo in patients not previously treated with statins.

## Materials and methods

### Study design

This was a single centre randomized, double-blind, parallel, placebo-controlled clinical trial. Ethical approval was granted by the South Birmingham Research Ethics Committee and the trial was registered with the International Standard Randomized Control Trial Registry (ISRCTN64637517). Valid informed written consent was obtained from all patients or their next of kin prior to enrolment in the trial.

### Study participants

Between June 2006 and August 2008, patients admitted to Birmingham Heartlands Hospital within 24 hours, with a white cell count (WCC) > 11 or < 4 × 10^9^/L and with C-reactive protein (CRP) 2 SD above the upper limit of normal (6 mg/L), were identified from the hospital's iCARE Vortal database. These patients were screened further for eligibility for enrolment into the trial.

The inclusion criteria [13] were as follows: age greater than 18 years; documented new proven or suspected infection, and the presence of any two of the signs and symptoms of infection (WCC > 11 or < 4 × 10^9^/L, temperature > 38°C or < 36°C, heart rate > 90/bpm, or respiratory rate > 20/minute) for less than 24 hours. We excluded patients with evidence of severe sepsis defined by the Surviving Sepsis Campaign Guideline (SSCG), that is, sepsis with failure of one or more organs [14]; pregnancy; acute or chronic liver failure; evidence of myopathy; rhabdomyolysis, or terminal illness. Prior statin therapy within 2 weeks, the concomitant use of macrolides, imidazoles, or other lipid-lowering therapy, and the inability to swallow also precluded enrollment.

An amendment was approved to allow enrolment of patients prescribed macrolides. These antibiotics form part of local guidelines for treatment of community-acquired pneumonia and are frequently prescribed. The additional risk of myopathy caused by competition for metabolism by cytochrome P450 3A4 between atorvastatin and macrolides was deemed to be mitigated by the careful monitoring of serum creatinine kinase (CK).

### Randomization and blinding

Study drug packs were prepared by DHP pharma (Powys, UK). The active and placebo drug components of capsules were packaged identically into numbered treatment packs, each containing 40 mg atorvastatin (Pfizer, Sandwich, UK) or placebo. We used a computer-generated randomization sequence with a block size of four. Patients were randomly assigned in a 1:1 ratio by a centralised randomization service (Heartlands Hospital Pharmacy, England, UK). Participants, care providers, and investigators were blinded to group assignment. Study drug was administered orally within 24 hours of randomization. Treatment continued for the duration of their hospital stay or 28 days if earlier. All other therapeutic decisions were at the discretion of the primary physician.

### Data collection

We obtained the data for diagnosis of sepsis and severe sepsis from medical notes and laboratory reports. Data was also collected to calculate Acute Physiology and Chronic Health Evaluation II (APACHE II) scores with its predicted hospital mortality and Modified Early Warning Scores (MEWS) to evaluate the severity of patients' conditions at baseline. We followed all participants up to 12 months after randomization and collected data for critical care admission rate, hospital readmissions at 28 days and 1 year, length of hospital stay, and 28-day and 1-year mortality. Quality of life (QOL) data were collected at baseline and day of discharge using the validated EuroQol visual analogue scale (see Additional file 1).

### Laboratory investigations

Appropriate microbiological samples were sent for culture to establish the site of infection and pathogen and collection was guided by clinical condition. Full blood count, plasma urea and electrolytes, liver function tests, CK levels, coagulation studies and arterial blood gas analysis were recorded. Lipid profiles and CRP were measured at baseline, day 4, day 7 and day 28. Urine samples were collected at the same intervals to measure urine albumin creatinine ratios (ACR).

### Study outcomes

The primary outcome was the progression rate of sepsis to severe sepsis during the hospital stay or by 28 days if earlier. The progression of sepsis to severe sepsis was identified using the SSCG screening tool as described in Table 1 (see Additional file 2). Secondary outcomes were critical care unit (CCU) admission rate, hospital readmission rate at 28 days and 1 year, length of hospital stay, and hospital 28-day and 1-year mortality.

**Table 1:** Criteria used for classifying organ dysfunction and progression to severe sepsis

Severe sepsis defined as new or suspected infection with one or more organ dysfunctions Organ dysfunction defined as:	
**Cardiovascular**	SBP < 90, MAP < 65 mmHg or > 40 mmHg fall in SBP from baseline and/or use of noradrenaline/dobutamine/dopamine
**Respiratory**	Bilateral pulmonary inflitrates with new or increased oxygen requirements to maintain oxygen saturations > 90% or bilateral pulmonary inflitrates with a PaO_2_/FiO_2 _ratio < 300 mmHg
**Renal**	Creatinine > 176.8 mmol/L or urine output < 0.5 mls/kg/hr for > 2 hrs
**Haematological**	Platelets < 100,000/mm^3^ INR > 1.5 or APTT > 60 secs
**Liver/metabolic**	Bilirubin > 34.2 mmol/L Lactate > 2 mmol/L

APTT: activated partial thromboplastin time; INR; international normalized ratio; MAP: mean arterial pressure; SBP: systolic blood pressure.

### Sample size and statistical analysis

Based on limited data available at the time [8], it was postulated that statins might reduce the absolute rate with which sepsis converted to severe sepsis by 15% (40% to 25% change). To detect such a reduction in risk, with a power of 80% and at the 5% level of significance, a total of 414 patients (*n *= 207 in each group) would be required. Based on the local and very limited European epidemiological data available at the time, it was expected that the trial would need 30 months for recruitment and 12 month for post-randomisation follow-up, making the total duration 42 months (3.5 years).

Data were analysed by an independent statistician on an intention-to-treat basis using SPSS for Windows 7. Data were tested for normality and analysed by unpaired *t*-tests or Mann Whitney *U*-test. Data are expressed as mean (SD) or median (interquartile range, IQR) as appropriate. The chi squared or Fisher's exact test was used to compare proportions. A *P*-value of 0.05 was considered significant.

## Results

**Figure 1:**
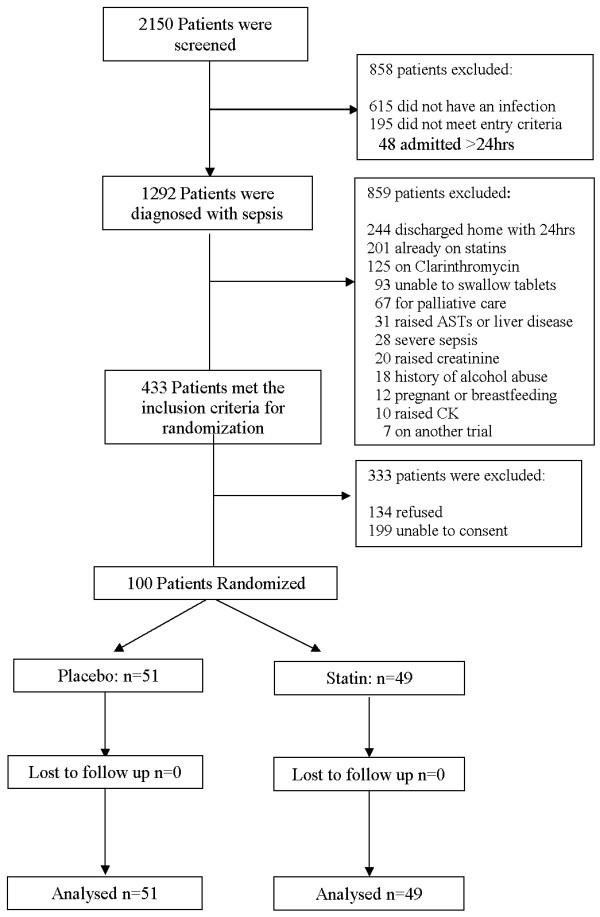
**Consort diagram illustrating the screening, enrolment and randomization of study patients**.

The screening for inclusion criteria was started in June 2006 and ended in August 2008 due to slow recruitment. During this period a total of 2,150 patients were screened; 1,292 (60%) of these were diagnosed with sepsis according to SSCG, and 433 (34%) met the inclusion criteria with 100 patients consenting to randomization (49 randomized to atorvastatin and 51 to placebo). No patients were lost to follow-up during their admission and all patient data were included in the analysis. Figure 1 shows the trial profile.

Patients in the two groups were well-matched in terms of the demographic data, biochemical presentations, global severity of illness (APACHE II and MEWS scores) and sources of sepsis (Table 2). Seven patients in the atorvastatin and thirteen in the placebo group had confirmed positive microbiology samples that isolated the sources of sepsis, (*P *= 0.21). Both groups received appropriate anti-microbial therapy (77% vs. 82%, *P *= 0.65) and received a similar number of trial drug doses (three vs. five, *P *= 0.32).

**Table 2:** Patients' baseline demographics and biochemical data

	Atorvastatin *n *= 49	Placebo *n *= 51	*P*-value
Age, years, mean (SD)	62.8 (21.2)	64 (15.6)	0.745
Male gender, %	51	51	0.570
**Scoring systems**			
MEWS, mean (SD)	2.8 (2.1)	3.3 (2.1)	0.197
APACHE II, mean (SD)	11.8 (5.6)	11.9 (6.0)	0.914
Predicted mortality, median (IQR)	14.6 (8.7, 23.5)	12.9 (7.6, 26.2)	0.909
**Biochemical data**			
Cholesterol, mmol/L, median (IQR)	4.0 (2.9, 5.1)	4.35 (3.35, 5.35)	0.505
Neutrophils, × 10^9^/L median (IQR)	11.6 (8.3, 13.7)	12.4 (9.3, 19.4)	0.232
C-reactive protein, mg/L, mean (SD)	194.6 (114.6)	188.0 (127.3)	0.683
**Source of infection, number of cases**			
Pneumonia	27	24	0.674
Urinary tract	8	9	0.808
Cellulitis	6	10	0.317
Gastrointestinal	5	7	0.563
Bacteraemia	2	1	0.563
Urinary catheter	1	0	0.317
**Antibiotic therapy, number of patients**	38	42	0.654
**Co-morbidities, number of patients**			
0	15	17	0.832
1	22	21	0.840
2	7	6	0.772
3 or more	5	8	0.554
**Number of trial drug doses, median (IQR)**	3 (2, 7)	5 (2, 8)	0.322

MEWS, modified early warning score; APACHE, acute physiology and chronic health evaluation; IQR, interquartile range.

### Primary outcome

The atorvastatin group had a significantly lower incidence of sepsis converting to severe sepsis (*n *= 2 patients) compared to the placebo group (*n *= 12) (4% vs. 24%, *P *= 0.007, number needed to treat = 5). Patients progressing to severe sepsis predominantly had respiratory failure (*n *= 7) with one patient requiring mechanical ventilation and one requiring inotropic support. Three patients in the placebo group developed failure of more than one organ, compared with one patient in the atorvastatin group (Table 3).

**Table 3:** Organ failure in the atorvastatin and placebo groups

	Atorvastatin	Placebo
Respiratory failure without mechanical ventilation	0	7
Respiratory failure with mechanical ventilation	0	1
Cardiovascular failure without inotropes	1	0
Cardiovascular failure with inotropes	0	1
Renal failure without RRT	0	0
Neurological	0	0
Failure of more than one organ	1	3
Totals	2	12

Results are presented as number of patients. RRT: renal replacement therapy.

### Secondary outcomes

The 28-day mortality was 4% with two deaths in each group (*P *= 1.0), while 1-year mortality was 8% (four patients in the atorvastatin group vs. four in the placebo group, *P *= 1.0), making overall mortality for the cohort 12% (Table 4). Median length of hospital stay for the atorvastatin and placebo groups was five days (IQR 3 to 13) and six days (IQR 4 to 12) respectively (*P *= 0.59). There was no effect on rates of CCU admission between the groups (*n *= 0 vs. 2 patients, *P *= 0.495). Of the 96 survivors, 28-day readmission data were available for 89 (92.7%). There were ten sepsis-related readmissions, with five in each group (*P *= 1.0). At 1 year 89 (89%) patients had survived and readmission data were available for 88 (98.9%) with no significant difference between the groups for the number of readmissions (*P *= 0.541).

**Table 4:** Length of stay, readmission and mortality data

	Atorvastatin *n *= 49	Placebo *n *= 51	*P*-value
Length of hospital stay, median (interquartile range)	5 (3, 13)	6 (4, 12)	0.598
Readmissions, number			
Within 28 days	5	5	1.0
	(*n *= 43)	(*n *= 46)	
Within 1 year	7	5	0.541
	(*n *= 42)	(*n *= 45)	
Mortality, number of patients			
28 days	2	2	1.0
1 year	4	4	1.0

QOL data assessed by the EuroQol visual analogue scale showed a significant increase in mean score from baseline to discharge for the cohort (45.2 vs. 65.4, 95% confidence interval (CI) 37.0, 72.3, *P *< 0.0001) representing a perceived improvement in QOL. No difference was observed between the two groups in median score at baseline (50.0 vs. 40.0, IQR 20 to 60, *P *= 0.51). At discharge, however, the placebo group had a better perceived median QOL score than the atorvastatin group (60.0 vs. 77.5, IQR 51.3 to 78.8, *P *= 0.02).

The change from baseline in lipid profiles for the two groups is shown in Table 5. No differences in baseline plasma cholesterol, high-density lipoprotein (HDL), low density lipoprotein (LDL) or triglycerides were observed between the groups. At day 4 a significant decrease was observed in plasma cholesterol and LDL (*P *< 0.0001 and 0.019 respectively) in the atorvastatin group compared to placebo. No difference in HDL or triglycerides was seen (*P *= 0.442 and 0.210 respectively).

**Table 5:** Median lipid profiles in the placebo and atorvastatin groups at day 4 of follow-up compared to baseline

	Placebo Difference from day 1 *n *= 31/31*	Atorvastatin Difference from day 1 *n *= 23/25*	Comparison atorvastatin-placebo	*P*-value
Total cholesterol (mmol/L)	0.30	-0.50	-0.8 (-0.9, -0.8)	< 0.0001
LDL(mmol/L)	0.16	-0.63	-0.79 (-0.93, -0.47)	0.019
HDL (mmol/L)	-0.18	-0.40	-0.22 (-0.53, 0.01)	0.227
Triglycerides (mmol/L)	0.50	0.20	-0.30 (-1.0, 0.40)	0.79

*Number of samples available for analysis. Denominator represents number of patients still in hospital at day 4. Values represent median and IQR. LDL, low density lipoprotein; HDL, high density lipoprotein.

**Figure 2:**
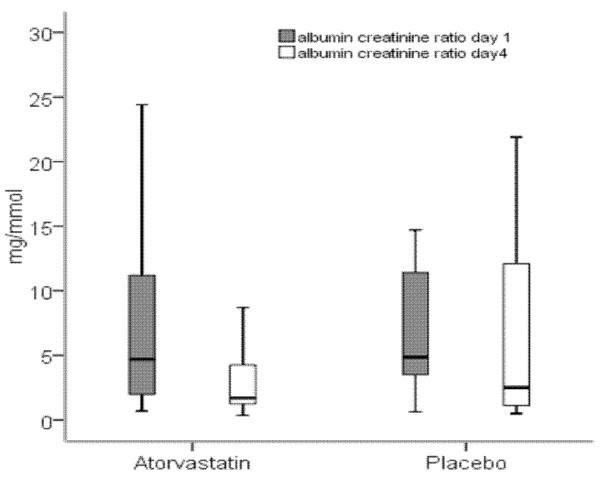
**Albumin creatinine ratio (ACR) at day 1 and day 4 in the atorvastatin and placebo groups**. Boxes represent the interquartile range; horizontal line represents the median; whiskers represent minimum and maximum values.

No difference in ACR was observed between the groups at day 1 or day 4. A significant reduction in albumin excretion in the atorvastatin group was seen between days 1 and 4 compared to placebo (*p *= 0.049 vs. 0.796, Figure 2).

### Adverse events

Serial blood samples were taken to monitor any serious adverse events due to statin therapy. There were no cases of raised transaminase levels in either group. There were, however, two patients with raised CK above 1,400 U/L in the atorvastatin group (*P *= 0.238). Both patients were diagnosed with acute coronary syndrome and the CK rise was attributed to this. There were no episodes of myalgia.

## Discussion

To our knowledge, this randomized control trial is the first to investigate the impact of the acute administration of statins on progression of sepsis in statin-naïve individuals. It demonstrates that statins were well absorbed in patients with sepsis, and administration early in the septic process significantly reduced the likelihood of sepsis progressing to severe sepsis. One case of severe sepsis was prevented for every five patients with sepsis.

This randomized controlled trial supports previous retrospective and prospective observational studies demonstrating that patients who are on pre-existing statin therapy have better sepsis-related outcomes compared to matched controls [4,6,8].

The significant reduction in urinary ACR at day 4 in the statin-treated group may explain slowing of sepsis progression in that group. Albumin excretion in urine is tightly regulated by the kidneys. In inflammatory states, especially sepsis, however, glomerular permeability increases due to altered endothelial function, resulting in increased protein excretion. This is an early sign in sepsis and persists in patients who develop organ dysfunction. We postulate that the significant reduction seen in ACR in the statin group may reflect less damage in endothelial function and hence a reduction in the progression of sepsis [15,16].

The atorvastatin group showed significant decreases in both plasma cholesterol and LDL between days 1 and 4. This demonstrates compliance with treatment, absorption of statins and effective plasma concentrations being achieved to cause decreases in plasma lipids. The ability of atorvastatin to reduce lipid levels after only three doses in combination with the reduced rates of sepsis progression seen would indicate that statins are able to exert their pleiotrophic effects acutely, thereby modulating the immune and cellular responses to sepsis. The HARP study showed that 80 mg of simvastatin administered acutely to patients with acute lung injury showed significant improvements in organ failure scores, suggesting that statins modulate the systemic inflammation after only a short duration of treatment [17].

EuroQol scores at baseline were low for the cohort indicating that the majority of patients considered their QOL to have deteriorated substantially from health due to their sepsis-related hospital admission. This is not surprising; however, when compared to their physiological and biological markers of infection, such as APACHE II scores, which were low, the EuroQOL scores indicate that although our patients had relatively mild disease they perceived their illness to have had a significant impact on their QOL. Reassuringly, the EuroQol scores had improved significantly by the time of discharge, suggesting that patients had improved not only in terms of bio-physical markers of disease but also in their personal well-being.

Although the trial did not achieve its recruitment target, it is still the second largest RCT to date investigating the role of statins in sepsis, and is the first to suggest benefit from acute statin therapy. The screening process involved assessing all admissions to the hospital on the basis of laboratory levels of CRP, WCC and systemic inflammatory response syndrome (SIRS) criteria. This process was neither specific nor sensitive for identifying patients with sepsis, and a high refusal rate was seen among those who were subsequently eligible for randomization. The majority of patients randomized were not critically ill, as observed by their low APACHE II scores at baseline. This supports previous studies that have encountered similar recruitment problems when conducting sepsis-related research on ward-based patients, as the vast majority are not critically ill at admission, have a low rate of progression of sepsis, and the diagnosis of sepsis remains non-specific and non-sensitive [9,10]. Recent validation studies using the predisposition, insult, response, organ dysfunction (PIRO) model for stratification of risk of sepsis have suggested that this may allow more sensitive and specific phenotyping of patients with sepsis. Future studies into sepsis should consider using such models as opposed to the classical criteria used in this study to increase the likelihood of targeting desired populations [18].

## Conclusions

In conclusion, our study is the first RCT to demonstrate a benefit from acute administration of atorvastatin 40 mg daily for preventing the progression of sepsis in statin-naïve individuals. We postulate that statins may modulate the pathophysiology of sepsis via a novel cellular mechanism thereby restoring endothelial integrity and thus blocking one of the mechanisms in the development of multi-organ failure. Future large-scale multi-centre RCTs are required to investigate whether these results are reproducible, further explore the role of statins in sepsis and elucidate the cellular mechanisms by which statins confer protection in sepsis.

## Key messages

• Atorvastatin 40 mg per day may prevent the progression of sepsis to severe sepsis in hospitalized patients.

• Statins may act acutely to prevent organ dysfunction.

• Cellular mechanisms of organ protection need further evaluation, which may be associated with the improvement of endothelial integrity.

• Larger RCTs are warranted to establish whether statins can to be used in the early phases of sepsis to prevent the onset of organ failure.

## Abbreviations

ACR: albumin creatinine ratio; APACHE II: acute physiology and chronic health evaluation; APTT: activated partial thromboplastin time; CI: confidence interval; CK: creatinine kinase; CCU: critical care unit; CRP: C-reactive protein; HDL: high-density lipoprotein; HMG-CoA: 3-hydroxy-3 methylglutaryl coenzyme A; IL-6: interleukin-6; INR: international normalized ratio; IQR: inter-quartile range; LDL: low density lipoprotein; MAP: mean arterial pressure; MEWS: modified early warning score; PIRO: predisposition, insult, response, organ dysfunction; QOL: quality of life; RCT: randomized controlled trial; rhAPC: recombinant activated protein C; RRT: renal replacement therapy; SBP: systolic blood pressure; SIRS: systemic inflammatory response syndrome; SSCG: Surviving Sepsis Campaign Guidelines; TNF-α: tumour necrosis factor-alpha; WCC: white cell count.

## Competing interests

This trial received funding from The Moulton Charitable Foundation and Pfizer Global Pharmaceuticals.

## Authors' contributions

FG was chief investigator for this study and takes full responsibility for the integrity of the data analysis. FG, DRT, GDP, MC, AJ, AB and PH were involved in the conception and design of the study. Data acquisition was performed by LL, TH and TM. The data analysis and interpretation was carried out by JMP, CS and DRT. The drafting of the manuscript was carried out by JMP and CS. Critical revisions of the manuscript were carried out by JMP, FG, GDP and DRT. All authors have read and approved the final manuscript for publication.

## Supplementary Material

Additional file 1 **EUROQOL II Score**. This was the visual analogue score part of the EUROQOL II score that was used to assess quality of life. Click here for file

Additional file 2 **Surviving Sepsis Campaign Screening Tool**. This was the validated screening tool used to identify patients with sepsis and those that progressed to severe sepsis. Click here for file
