# Supplementary material for: Randomized double-blind placebo-controlled trial of 40 mg/day of atorvastatin in reducing the severity of sepsis in ward patients (ASEPSIS Trial)
Source: Crit Care. 2012 Dec 11;16(6):R231. doi: 10.1186/cc11895 (PMC3672620; doi:10.1186/cc11895)
Supplement: Additional file 1 — EUROQOL II Score. This was the visual analogue score part of the EUROQOL II score that was used to assess quality of life. [file cc11895-S1.DOCX]

**Appendix 2:** EuroQol Visual Anlogue Scale

| **EuroQol - Health related quality of life assessment II** | | | | | | | | | | |
| --- | --- | --- | --- | --- | --- | --- | --- | --- | --- | --- |
|  |  |  |  |  |  |  |  |  |  |  |
|  | | |  |  |  |  |  |  |  |  |
|  |  |  |  |  |  |  |  |  |  |  |
| To help people to say how good or bad a health state is, we have drawn | | | | | | | | |  |  |
| a scale (rather like a thermometer) on which the best state you can imagine is marked by 100 | | | | | | | | | | |
| and the worst state you can imagine is marked by 0. | | | | |  |  |  |  |  |  |
|  |  |  |  |  |  |  |  |  |  |  |
| We would like you to indicate on this scale how good or bad is your own health today, | | | | | | | | | | |
| in your opinion. Please do this by drawing a line from the box below to whichever point | | | | | | | | | | |
| on the scale indicates how good or bad your current health state is. | | | | | | | |  |  |  |
|  |  |  |  |  |  |  |  |  |  |  |
|  |  |  |  |  |  |  |  |  |  |  |
|  |  |  |  | Best imaginable |  |  |  |  | 100 |  |
|  |  |  |  | health state |  |  |  |  |  |  |
|  |  |  |  |  |  |  |  |  |  |  |
|  |  |  |  |  |  |  |  |  | 90 |  |
|  |  |  |  |  |  |  |  |  |  |  |
|  |  |  |  |  |  |  |  |  |  |  |
|  |  |  |  |  |  |  |  |  | 80 |  |
|  |  |  |  |  |  |  |  |  |  |  |
|  |  |  |  |  |  |  |  |  |  |  |
|  |  |  |  |  |  |  |  |  | 70 |  |
|  |  |  |  |  |  |  |  |  |  |  |
|  |  |  |  |  |  |  |  |  |  |  |
|  |  |  |  |  |  |  |  |  | 60 |  |
|  |  |  |  |  |  |  |  |  |  |  |
|  |  |  |  |  |  |  |  |  |  |  |
|  |  |  |  |  |  |  |  |  | 50 |  |
|  |  |  |  |  |  |  |  |  |  |  |
|  |  |  |  |  |  |  |  |  |  |  |
|  |  |  |  |  |  |  |  |  | 40 |  |
|  |  |  |  |  |  |  |  |  |  |  |
|  |  |  |  |  |  |  |  |  |  |  |
|  |  |  |  |  |  |  |  |  | 30 |  |
|  |  |  |  |  |  |  |  |  |  |  |
|  |  |  |  |  |  |  |  |  |  |  |
|  |  |  |  |  |  |  |  |  | 20 |  |
|  |  |  |  |  |  |  |  |  |  |  |
|  |  |  |  |  |  |  |  |  |  |  |
|  |  |  |  |  |  |  |  |  | 10 |  |
|  |  |  |  |  |  |  |  |  |  |  |
|  |  |  |  |  |  |  |  |  |  |  |
|  |  |  |  | Worst imaginable |  |  |  |  | 0 |  |
|  |  |  |  | health state |  |  |  |  |  |  |

Reprinted with Permission: 1990 EuroQol Group EQ-5D™ is a trade mark of the EuroQol Group
