# Supplementary material for: Randomized double-blind placebo-controlled trial of 40 mg/day of atorvastatin in reducing the severity of sepsis in ward patients (ASEPSIS Trial)
Source: Crit Care. 2012 Dec 11;16(6):R231. doi: 10.1186/cc11895 (PMC3672620; doi:10.1186/cc11895)
Supplement: Additional file 2 — Surviving Sepsis Campaign Screening Tool. This was the validated screening tool used to identify patients with sepsis and those that progressed to severe sepsis. [file cc11895-S2.PDF]

*Chart record – use patient label.  Do not remove from chart*

# Evaluation for Severe Sepsis Screening Tool

**Instructions: Use this optional tool to screen patients for severe sepsis in the emergency department, on the wards, or in the ICU.**

**1. Is the patient's history suggestive of a new infection?**

- ☐ Pneumonia, empyema
- ☐ Urinary tract infection
- ☐ Acute abdominal infection
- ☐ Meningitis
- ☐ Skin/soft tissue infection
- ☐ Bone/joint infection
- ☐ Wound infection
- ☐ Bloodstream catheter infection
- ☐ Endocarditis
- ☐ Implantable device infection
- ☐ Other ____________

**___ Yes ___No**

**2. Are any two of following signs & symptoms of infection both present and new to the patient? Note: laboratory values may have been obtained for inpatients but may not be available for outpatients.**

- ☐ Hyperthermia > 38.3 °C (101.0 °F)
- ☐ Hypothermia < 36 °C (96.8°F)
- ☐ Tachycardia > 90 bpm
- ☐ Tachypnea > 20 bpm
- ☐ Acutely altered mental status
- ☐ Leukocytosis (WBC count >12,000 µL–1)
- ☐ Leukopenia (WBC count < 4000 µL–1)
- ☐ Hyperglycemia (plasma glucose >120 mg/dL) in the absence of diabetes

**___ Yes ___No**

**If the answer is yes to both either question 1 and 2, *suspicion of infection* is present:**

- ✓ Obtain: **lactic acid**, **blood cultures**, CBC with differential, basic chemistry labs, bilirubin.
- ✓ At the physician's discretion obtain: UA, chest x-ray, amylase, lipase, ABG, CRP, CT scan.

**3. Are any of the following organ dysfunction criteria present at a site remote from the site of the infection that are not considered to be chronic conditions? Note: the remote site stipulation is waived in the case of bilateral pulmonary infiltrates.**

- ☐ SBP < 90 mmHg or MAP < 65 mmHg
- ☐ SBP decrease > 40 mm Hg from baseline
- ☐ Bilateral pulmonary infiltrates with a new (or increased) oxygen requirement to maintain SpO2 > 90%
- ☐ Bilateral pulmonary infiltrates with PaO2/FiO2 ratio < 300
- ☐ Creatinine > 2.0 mg/dl (176.8 mmol/L) or Urine Output < 0.5 ml/kg/hour for > 2 hours
- ☐ Bilirubin > 2 mg/dl (34.2 mmol/L)
- ☐ Platelet count < 100,000
- ☐ Coagulopathy (INR >1.5 or aPTT >60 secs)
- ☐ Lactate > 2 mmol/L (18.0 mg/dl)

**___ Yes ___No**

**If *suspicion of infection* is present AND *organ dysfunction* is present, the patient meets the criteria for SEVERE SEPSIS and should be entered into the severe sepsis protocol.**

Date: ____/____/____ (circle: dd/mm/yy or mm/dd/yy)    Time: ____: ____ (24 hr. clock)

Version 7.12.2005

© 2005 Surviving Sepsis Campaign and the Institute for Healthcare Improvement
